# Experimental Investigation of Temperature and Contact Pressure Influence on HFI Welded Joint Properties

**DOI:** 10.3390/ma15103615

**Published:** 2022-05-18

**Authors:** Christian Egger, Martin Kroll, Kerstin Kern, Yannik Steimer, Michael Schreiner, Wolfgang Tillmann

**Affiliations:** 1 Institute for Computational Engineering (ICE), Eastern Switzerland University of Applied Sciences, 9471 SG Buchs, Switzerland; michael.schreiner@ost.ch; 2 Institute of Materials Engineering (LWT), Technical University of Dortmund, 44227 Dortmund, Germany; wolfgang.tillmann@tu-dortmund.de; 3 Institute for Machine Tools and Production Processes (IWP), Chemnitz University of Technology, 09107 Chemnitz, Germany; martin.kroll@mb.tu-chemnitz.de; 4 Institute for Microtechnology and Photonics (IMP), Eastern Switzerland University of Applied Sciences, 9471 SG Buchs, Switzerland; kerstin.kern@ost.ch (K.K.); yannik.steimer@ost.ch (Y.S.)

**Keywords:** high-frequency welding, pressure welding, weld seam strength, weld seam characterization, weld seam microstructure, welding temperature, upsetting force

## Abstract

This paper presents an experimental electro-thermo-mechanical simulation of high-frequency induction (HFI) welding to investigate the effect of temperature and contact normal stress on the weld seam quality. Therefore welding experiments at different temperatures and contact pressures are performed using flat specimens of 34MnB5 steel sheet. In order to characterize the weld seam strength of the welded specimens, tensile and bending tests are performed. To obtain a relative weld seam strength, the bending specimens were additionally hardened prior to testing. With the hardened specimens, it can be shown that the weld seam strength increases with increasing temperature and contact normal stress until a kind of plateau is formed where the weld seam strength remains almost constant. In addition to mechanical testing, the influence of the investigated process parameters on the weld seam microstructure is studied metallographically using light optical microscopy, scanning electron microscopy, EBSD and hardness measurements. It is shown that the weld seam strength is related to the amount of oxides in the bonding line.

## 1. Introduction

The weight of cars can be reduced by replacing solid cylindrical components by high-strength steel tubes. In cars with conventional engines, this leads to reduced fuel consumption and CO${}_{2}$ emissions [1]. In electrical vehicles, lightweight constructions can help to extend range [2]. Because of economical reasons, steel tubes are typically manufactured by longitudinal welding. The most common longitudinal welding technique is high-frequency induction (HFI) welding. In this process, a steel band is formed into an open seam tube by roll forming. Afterwards, the band edges are heated by electromagnetic induction and the remaining slit is closed by press welding.

The steel components used in the automotive industries are often manufactured of manganese boron steels such as 34MnB5 with a relatively high carbon and manganese content. Such steel grades are well suited for quenching or press hardening, but difficult to weld. The difficulties are mainly caused by cold cracking, which can occur due to the hardening of the heat-affected zone in combination with residual and load stresses [3]. Due to the absence of filler metals, pressure welding processes, such as HFI welding of steels, are usually carried out with grades up to 0.22 wt% carbon to ensure good weldability [4]. For pipeline transport systems, the carbon content is limited to 0.23 wt% and the carbon equivalents (CE) to CE${}_{\mathrm{IIW}}$ = 0.43% according the International Institute of Welding [5,6]. With a carbon content of 0.33 wt% and a carbon equivalent of CE${}_{\mathrm{IIW}}$ = 0.58%, 34MnB5 is expected to have poor weldability when considering only the carbon content and CE. Unfortunately, it is not feasible to measure the quality or the strength of the weld seam in the production line. Thus, the quality of the weld seam can only be assessed based on process parameters. For this purpose, the influence of the process parameters on the weld seam quality has to be known. However, this correlation is hard to determine on a tube welding line. One possibility is to simulate the HFI welding process on an conductively heated electro-thermo-mechanical test rig with flat specimens. An advantage of this experimental approach is that independent process parameters such as the upsetting distance, the effective welding power, the welding frequency and the welding speed can be adjusted separately in a systematic manner. In addition, dependent process parameters such as the welding temperature, the temperature distribution and the upsetting force can easily be measured. Further, the strength of the weld seam can be analyzed based on simple tension- and bending-tests.

A bond between two bodies can be achieved by applying heat, pressure or a combination of both [7,8]. Depending on the values of these two parameters, a classification between solid state welding, diffusion welding and melting with solidification can be made [9]. In HFI welding, the heat is generated directly in the steel band by induced eddy currents. Due to the skin and proximity effect, the band edges are heated only locally. Afterward, the heated band edges are pressed against each other, squeezing molten material, oxides and impurities out of the weld area [10,11]. Due to the combined effect of high temperatures and pressures, the HFI welding process can be characterized as a mixture of solid state welding, diffusion welding and melting with solidification. According to Messler [12], the temperature and the contact pressure have a large effect on the welded joint properties.

Weldability as a function of upsetting pressure, temperature and surface condition was investigated for pure iron [13,14]. It was found that the weld strength increased steadily with higher temperature. Using welded tubes, Förster [15] was able to show that imperfect welds are caused by too low welding temperatures or upsetting pressures. Using a Gleeble^®^ simulator (Dynamic Systems Inc., Poestenkill, NY, USA), it was shown that temperatures close to the liquidus temperature are necessary in tube welding [16]. This paper also proposes a mechanism for joint formation during pressure welding. The mechanism of bonding line formation and further metallographic studies to characterize the weld seam are discussed in many other publications [17,18,19,20,21,22]. However, the effect of different temperatures and contact normal stresses on weld seam strength and microstructure formation in HFI welding has never been considered in a scientific publication.

The objective of this work is to investigate the influence of the temperature and the contact normal stress on the weld seam quality of high-frequency induction welded seams. An electro-thermo-mechanical welding rig was used to selectively vary the temperature and upsetting force. By means of light optical microscopy, scanning electron microscopy, electron backscatter diffraction and hardness measurements, the influence of the process parameters on the weld seam was investigated metallographically. Furthermore, tensile and bending tests were used to characterize the mechanical properties of the weld seam. Based on the mechanical test results and the process data, a correlation between the temperature, the contact normal stress and the weld seam strength ratio was found. The established correlation can be used for the optimization of HFI tube welding processes. Furthermore, the obtained relationship can be used in finite element (FE) simulations to estimate the weld seam quality based on the calculated temperatures and contact normal stresses.

## 2. Materials and Methods

In this section, the material properties of the steel used in this study are described. In addition, the electro-thermo-mechanical simulator used to simulate the HFI longitudinal tube welding process is presented. Furthermore, the microstructural analysis and the mechanical tests performed to characterize the weld seam properties are described and the weld seam quality is introduced.

### 2.1. Material

The material used in this study is a batch annealed 34MnB5 steel sheet with a thickness of 3.5 mm. The chemical composition, as measured by glow discharge optical emission spectroscopy (GDOES) with a GDA 150 HR (Spectruma Analytik GmbH, Hof, Germany), is shown in Table 1. Concerning the mechanical properties the most relevant alloying elements are carbon, manganese and boron.

### 2.2. Electro-Thermo-Mechanical HFI Tube Welding Simulator

**Figure 1:**
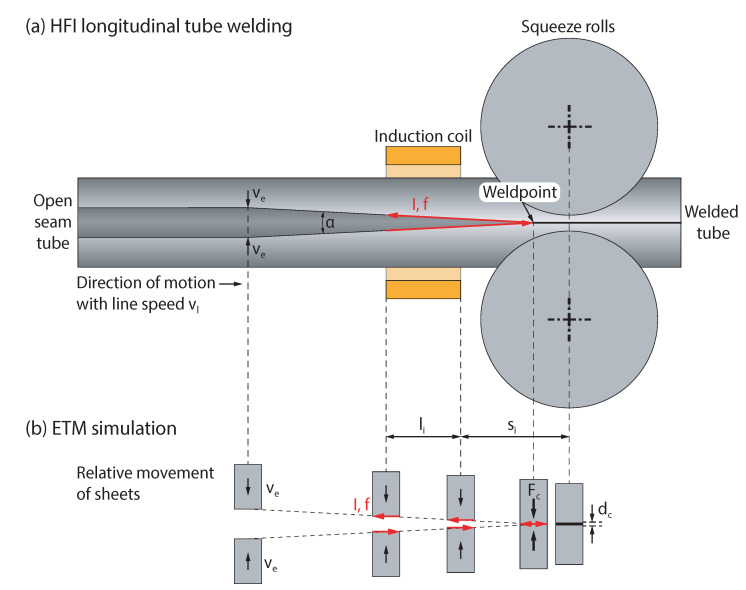
Comparison of (**a**) the HFI longitudinal tube welding process with (**b**) the experimental ETM simulation.

The experimental welding was performed on an electro-thermo-mechanical (ETM) conductively heated tube welding simulator (Figure 1) that was developed and realized at Chemnitz University of Technology. The purpose of this device is to replicate the continuous HFI longitudinal tube welding process of steel bands (Figure 1a) with a line speed $v_{l}$. Therefore, two single steel sheets are moved towards each other with the speed $v_{e}$ in an experimental scale (Figure 1b). In contrast to the electromagnetic induction of eddy currents in HFI welding, the alternating current in the ETM simulator is applied by direct electrical contacting of two separate sample sheets, representing both band edges in the industrial process. The propagation of the two band edges towards each other with a defined relative speed $v_{e}=f\left(v_{l},\alpha \right)$ up to the weldpoint in the industrial tube welding process is represented by the relative movement of both sample sheets in the ETM simulation $v_{e}$. In HFI tube welding, the band edges are subjected to heating caused by resistive heating of induced eddy currents during the propagation of the formed steel band from the induction coil towards the weldpoint. This joule heating and the resulting heat distribution is reproduced in the ETM simulation. The subsequent pressure welding of the heated band edges is performed by pressing the heated sheets together with a defined maximum upsetting force $F_{c}$, which leads to a compression distance $d_{c}$. This pressure welding process produces weld excess in the form of a weld bead. By the squeeze out of molten and semi-molten material, the oxides, that have been formed during the heating of the band edges, are expelled from the weld junction. In summary, the ETM simulation is able to represent the material, the wall thickness *s*, the entry or welding angle α, the line speed $v_{l}$ and derived movement of the band and sheet edges $v_{e}=f\left(v_{l},\alpha \right)$, the position $s_{i}$ and length $l_{i}$ of the induction coil, the welding frequency *f*, the welding temperatures and the temperature distribution of the HFI longitudinal tube welding process.

The ETM simulator is comprised of an HF inverter in the form of an SDF^®^ 225 Simultaneous Dual Frequency generator (EMAG eldec Induction GmbH, Dornstetten, Germany) as the AC energy source. It provides up to 150 kW AC current at frequencies of f= 140–350 kHz. This energy source corresponds to a HF welder in industrial tube mills for longitudinal welding of tubes with wall thicknesses between 2–10 mm. The movement of the sheets and the directly following pressing process in the ETM simulator is facilitated by synchronized servo drives. The surface temperature was measured with an PI 640 thermal camera (Optris GmbH, Berlin, Germany) with an 33 ° × 25 °/f = 18.7 mm optic. Continuous force measurements were performed by a 9051A piezoelectric force sensor (Kistler Group, Winterthur, Switzerland) at f= 50 Hz during process. The AC power source and the mechanical drives are controlled centrally.

The welding experiments simulated the longitudinal welding of a 60 × 3.5 mm tube with a welding speed of $v_{l}=$ 20 m/ min at a welding frequency of f= 213–223 kHz. Therefore, an alternating current range of $I_{\mathrm{pp}}=$ 4115–4767 A, corresponding to a power range of P= 72–134 kW was applied in the stated frequency range. The applied upsetting force ranged from $F_{c}=$ 4.5–17.8 kN, which resulted in contact normal pressures $p_{c}$ of about 30–100 MPa and compression distances of $d_{c}=$ 0.1–1.4 mm.

### 2.3. Microstructural Analysis

Metallographic cross sections were prepared from the welded sheets to analyze the microstructure, defects in the bonding line and the hardness of the material within the heat affected zone. The cross sections were etched with a modified Beraha etchant ( 3 g potassium metabisulfite in 100 mL water) or with a 3% nital solution prior to the microscopy analysis. The latter was performed in brightfield with an optical microscope Axioskop 2 MAT (Carl Zeiss AG, Wetzlar, Germany). A JSM-IT800 scanning electron microscope (JEOL Ltd., Akishima, Japan) was used to examine the microstructure and for electron dispersive spectroscopy measurements. Electron backscatter diffraction (EBSD) analysis was performed with a DigiView 5 EBSD Camera (EDAX, Mahwah, NJ, USA). The parent grain reconstruction was performed with the software OIM Analysis 8.6 (EDAX, Mahwah, NJ, USA) and the Kurdjumow-Sachs orientation relationship [23]. The hardness measurements were carried out according to DIN EN ISO 6507-1 with a test load of 0.4903 N (=HV 0.05) on a hardness testing machine of the type DuraScan 50 (ZwickRoell GmbH, Ulm, Germany). Nanoindentation hardness mappings of the bonding line were performed using an FT-104A Femto-Indenter (FemtoTools AG, Buchs ZH, Switzerland) according to ISO 14577. The measurements were made with a Berkovich tip, a hardness indentation spacing of 1.0 μm, and a displacement controlled indentation depth of 100 nm.

### 2.4. Mechanical Testing

**Figure 2:**
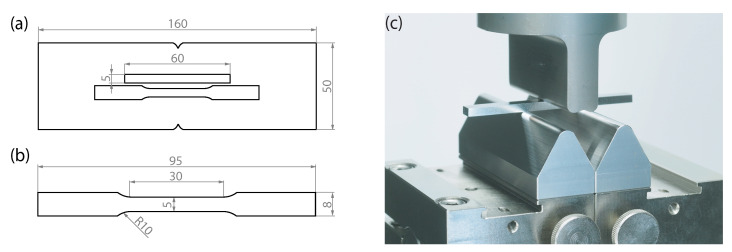
(**a**) Extraction of the specimens with thickness 2.8 mm for the mechanical tests from the welded sheets with the dimensions of the bending sample, (**b**) Dimension of the tensile specimen, (**c**) Test setup for performing the bending tests.

**Figure 3:**
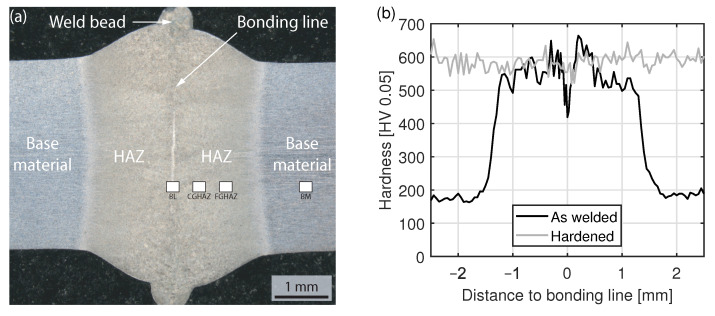
(**a**) Optical image of the as-welded sheet, etched with a 3% nital solution, (**b**) Hardness profile in the as-welded and hardened state.

**Figure 4:**
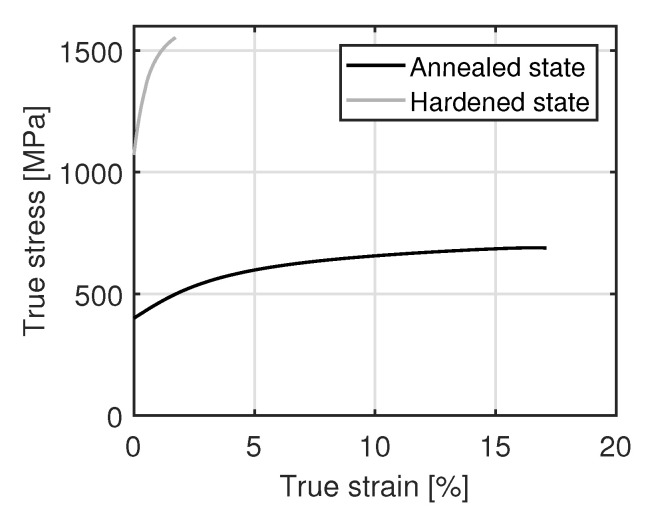
Flow curves of annealed and hardened 34MnB5 steel.

In order to analyze different mechanical test methods and material conditions, simulations were performed with the FEM software Ansys Mechanical (ANSYS, Inc., Canonsburg, PA, USA). The specimen geometries used for the simulation are identical to the tensile and bending specimens in the experiment (Figure 2). In the tensile test, the hard weld seam was modeled with a width of 3.0 mm, as shown in the hardness profile in Figure 3b. In the bending test, the material properties of the hardened state were used for the complete specimen, since there is no difference in hardness between the base material and the weld seam. The material behavior of the steel was modeled elastic-plastic. Therefore, tensile tests according to DIN EN ISO 6892-1, Control Method A1 were performed to determine the stress–strain curve. The true stress–strain behaviour in Figure 4 was approximated by the equations

(1) $$\varepsilon_{t}=\int_{l_{0}}^{l_{1}}\frac{1}{l}\;dl=ln\left(1+\varepsilon_{\mathrm{eng}}\right)$$

(2) $$\sigma_{t}=\frac{F}{A}=\sigma_{\mathrm{eng}}\left(1+\varepsilon_{\mathrm{eng}}\right)$$

that are valid up to tensile strength within the region of uniform elongation. Where $\varepsilon_{t}$ and $\sigma_{t}$ denote the true strain and stress, *F* is the acting normal force, *A* the current cross-section, *l* the current reference length, $l_{0}$ the initial length, while $\varepsilon_{\mathrm{eng}}$ and $\sigma_{\mathrm{eng}}$ denote engineering strain and stress. The required tensile specimens were taken from the steel band in the transverse direction according to DIN 50125–E5.5 × 10 × 42. In order to determine the material properties in the hardened state, some of the tensile specimens were austenitized at 900 °C for 20 min in a nitrogen atmosphere in a furnace and then quenched in oil to room temperature. The tensile specimens were tested on a Z100 universal testing machine equipped with a videoXtens 2-120HP (ZwickRoell GmbH, Ulm, Germany) at a forming rate of 0.0067 1/s and 0.00025 1/s up to yield strength. The specimens were preloaded with a stress of 10 MPa.

In order to determine the weld seam strength, one tensile and one bending specimen were taken from each welded plate by waterjet cutting, as shown in Figure 2. Prior to this, the weld bead was removed by grinding to obtain two parallel smooth surfaces. After that, the bending specimens were hardened. For this purpose, the specimens were austenitized at 900 °C for 20 min in a nitrogen atmosphere and then quenched in oil to room temperature.

The mechanical tests were performed using a universal testing machine Z100 (ZwickRoell GmbH, Ulm, Germany). Tensile specimens were tested and delivered typical stress–strain-curves according to DIN EN ISO 6892-1, Control Method A1. The test starts with a strain controlled rate of 0.00025 1/s up to the yield strength. Beyond the yield strength the strain rate is position controlled with a rate of 0.0067 1/s. The sample was pre-loaded with a stress of 3 MPa. The deformation of the sample was measured optically using a videoXtens 2-120HP. The initial gauge length of the sample was 25 mm.

A four-point-bending test, as shown in Figure 2, was carried out with the same testing machine. All supports had a radius of 3 mm. The support member were positioned with a support span of 20 mm, while the loading span of the loading member was 6 mm. The load rate during testing was position controlled with 2 mm/min. The displacement of the loading member was measured optically.

### 2.5. Weld Seam Strength Ratio

In general, mechanical tests of welded sheets or tubes measure the upsetting displacement or upsetting force. These measured quantities allow a comparison between the same sample sizes and the same material properties. However, it is difficult with these quantities to compare different welding parameters, test materials, specimen geometries and test types. Therefore, in this study, the weld seam strength ratio

(3) $$Q_{\mathrm{WS}}=\frac{\mathrm{strength}\;\mathrm{of}\;\mathrm{weld}\;\mathrm{seam}}{\mathrm{strength}\;\mathrm{of}\;\mathrm{base}\;\mathrm{material}}=\frac{F_{\mathrm{WS}}}{F_{\mathrm{BM}}}$$

is used to evaluate and compare the different test types and welding parameters. Where $F_{\mathrm{WS}}$ corresponds to the highest measured force on the specimen with weld seam and $F_{\mathrm{BM}}$ corresponds to the highest measured force on the specimen of the base material during the test. In the bending test, these forces usually correspond to the fracture force, whereas in the tensile test, these forces usually correspond to the ultimate tensile strength. Here, a weld seam strength ratio $Q_{\mathrm{WS}}$ of 1 means that the mechanical properties are the same in the weld seam as in the base material. If the two sheets are not welded together, due to insufficient temperature or contact normal stress, the weld seam strength ratio $Q_{\mathrm{WS}}$ is 0.

## 3. Results

In this section, the properties, the fracture forces and the calculated weld seam strength ratios $Q_{\mathrm{WS}}$ of the welded sheets are presented. Further, a correlation between the temperature, the upsetting force and the weld seam strength ratio $Q_{\mathrm{WS}}$ is obtained. It is also shown that there is a correlation between the mechanical properties and the oxide inclusions in the bonding line and on the fracture surface.

### 3.1. Microstructure and Hardness of the Welded Sheets

Due to the conductive heating of the band edges, the pressure welding and the subsequent cooling, the weld seam is subject to a process-, time- and location-dependent heat distribution, which locally affects the microstructure development, chemical composition and hardness. The weld seam can be divided into the bonding line, the heat affected zone (HAZ) and the weld bead, which consists of the liquid material squeezed out of the weld zone during pressure welding. An optical microscope image of a weld seam is shown in Figure 3a. Because of the heterogeneous microstructure in the welded state, the hardness drops from 600 HV0.05 to 200 HV0.05 in the transition zone from the HAZ to the base material (Figure 3b). In the bonding line, a second characteristic drop in hardness (420 HV0.05) can be observed. After hardening, the hardness in the entire sheet is about 590 ± 60 HV0.05. The fluctuation in hardness values can mainly be attributed to segregation in the steel.

#### 3.1.1. As-Welded Samples

In the as-welded state, the initial microstructure of the base material is present in all areas that are more than 2 mm away from the bonding line. The predominantly ferritic microstructure with cementites has an average grain diameter of 8.3 μm (Table 2) and a hardness of 190 HV. The adjacent fine grained heat affected zone (FGHAZ) consists of lath martensite with a prior austenite grain size (PAGs) of 11.8 μm. The martensite contributes to the increased hardness values between 510 HV and 650 HV in the HAZ. The PAG size increases with decreasing distance from the bonding line (BL). In the coarse grained heat affected zone (CGHAZ), lath martensite with a PAG size of 20.2 μm is present. The martensitic structure in the area of the bonding line has a PAG size of 34 μm. The grain size formed is related to the maximum austenitisation temperature and the holding time [24]. The maximum temperatures reached during welding decrease with increasing distance from the bonding line due to the skin and proximity effect [25]. For this reason, the largest grains form near the bonding line.

The drop in hardness in the bonding line is due to the reduced content of carbon, silicon and manganese, compared to the HAZ and the base material (Table 3) [26]. The carbon content can only be interpreted qualitatively due to the artificial carbon peak in the EDS measurement. On the one hand, the decrease in carbon in the bonding line results from a decarburization of the hot band edges in the welding process prior to their contact [27]. On the other hand, the carbon concentration in the liquid phase is higher than in the solid phase [28]. Due to the upsetting, the liquid, high-carbon phase is squeezed out of the welding zone, whereas the solid, low-carbon phase remains in the bonding line [22]. The decrease of manganese and silicon is attributed to the diffusion of the elements to the hot band edges. There, these elements react with atmospheric oxygen to form oxides such as MnSiO${}_{3}$ or Mn${}_{2}$SiO${}_{4}$ [29,30]. These oxides are then squeezed out of the welding zone with the melt. What remains is a bonding line with a reduced proportion of these alloying elements.

Due to the partially ferritic microstructure, the bonding line is sometimes also called ferrite line [16]. In Table 4, the bainitic ferrite with coarse, elongated site-plates is shown [31,32]. The specimens were etched with a modified Beraha etchant for 20 s. With this etching, only the areas with a high carbon content are etched [33]. This makes the ferritic structure with a low carbon concentration in the bonding line visible. Due to the body-centered cubic (bcc) structure and the strong drop in hardness in the bonding line, the presence of retained austenite can be ruled out. The only area with a face-centered cubic (fcc) structure is the titanium carbide near the bonding line.

#### 3.1.2. Hardened Samples

Specimens austenitised and quenched after welding at 900 °C for 20 min (Table 5) show a fully martensitic microstructure. Consequently, the hardness varies in a narrow range from 530 HV to 620 HV. The PAG size is 14 μm in the bonding line and increases to a diameter of 18 μm in the base material. The reason for the smaller grain size in the bonding line is the forming energy generated during welding, which results in nucleation points for new grain formation in this area.

### 3.2. Relationship between Welding Parameters and Weld Seam Strength Ratio $Q_{\mathrm{WS}}$

The two most commonly used methods for testing flat specimens are the tensile and bending tests, while testing specimens with different material properties, e.g., base material and weld seam, inhomogeneous stress and strain states can occur. In order to analyze these stress and strain states, simulations are performed using the finite element method (FEM). The flow curves of the annealed and hardened 34MnB5 steel used for the simulation are shown in Figure 4. Based on the FEM results, the test parameters, specimen geometries and material conditions of the specimens were defined.

**Figure 5:**
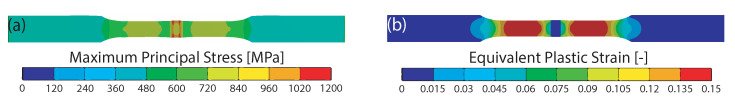
Finite element simulation of the tensile test with a hard weld seam located in the middle of the specimen and soft base material, (**a**) Maximum Principal Stress, (**b**) Equivalent Plastic Strain.

Flat specimens can be tested under uniaxial tensile loading according to DIN EN ISO 6892-1 and DIN 50125. This method is used in various publications to test welded specimens with hardened weld seams [17,34,35]. In almost all cases the fracture occurs outside the weld seam. Thereby, the measured ultimate tensile strength often exceeds the strength of the base material [36]. In the tensile test, the maximum stresses occur at the transition between the hard weld seam and the soft base material, as shown in Figure 5a. The maximum equivalent plastic strain occur about 10 mm next to the weld seam, as shown in Figure 5b. That is why necking and fracture will occur at this point.

**Figure 6:**
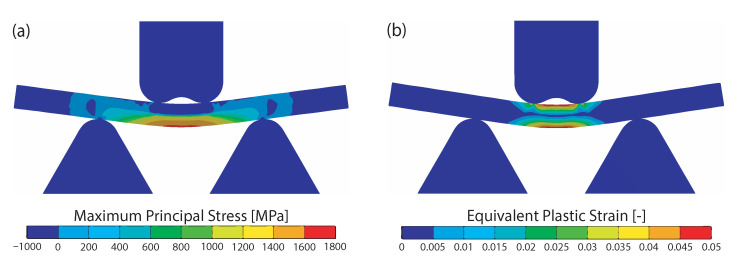
Finite element simulation of the bending test in the hardened state. The same material parameters have been used for the weld seam and the base material, (**a**) Maximum Principal Stress, (**b**) Equivalent Plastic Strain.

Due to the fact that the fracture of the specimens with a hard weld seam and a soft base material often occurs next to the weld seam, this specimen design is not suitable for a systematic investigation of different weld seam strength ratios. In order to reliably investigate the weld seam strength, homogeneous mechanical properties are required in the weld seam, the heat affected zone and the base material. This can be achieved by normalizing or hardening. Since the test should react as sensitively as possible to defects and should represent the condition of the final components as close as possible, hardening of the specimens is preferred for the investigated alloy. Metallic hardened specimens are commonly tested by bending. In the four-point bending test with hardened specimens, the highest stresses and strains occur in the area of the weld seam and the bonding line, as shown in Figure 6. Due to this load-characteristic, the described test is suitable for investigating the weld seam strength and for comparing different welding parameters.

In order to investigate the influence of different welding parameters on the weld seam strength ratio $Q_{\mathrm{WS}}$, 23 welding settings were used. These welding settings are given in Table 6 with the measured fracture forces $F_{\mathrm{WS}}$ and calculated weld seam strength ratios $Q_{\mathrm{WS}}$ of the tensile and bending specimens. The welding settings were varied in such a way that both the influence of the temperature and the influence of the contact normal stress on the weld seam strength ratio $Q_{\mathrm{WS}}$ could be investigated.

**Figure 7:**
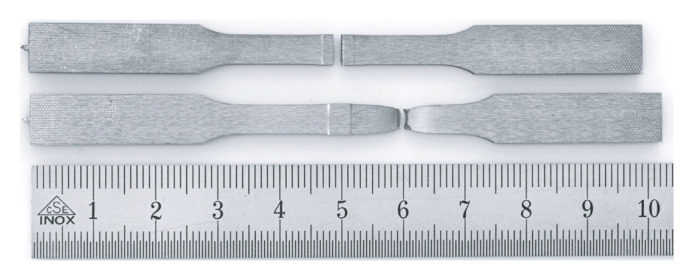
Fracture locations from the tested welded tensile specimens. Almost all tested tensile specimens break next to the weld seam, as shown below. Only, very weakly bonded specimens break in the bonding line, as shown above.

In the tensile test, almost all specimens in the welded condition fracture about 10 mm next to the hard weld seam, as shown in Figure 7. In case of tensile testing, the only two parameter combinations, that lead to a failure in the bonding line are at very low welding power of 81 kW. Due to this low power only a welding temperature of 1320 °C was achieved. This is the lowest temperature at which welding of the two sheets is still possible. Consequently only the worst specimens are found by the tensile test. A more detailed analysis of the weld seam strength ratio $Q_{\mathrm{WS}}$ can be made with the hardened specimens in the bending test. In the bending test, all hardened samples fracture in the bonding line.

**Figure 8:**
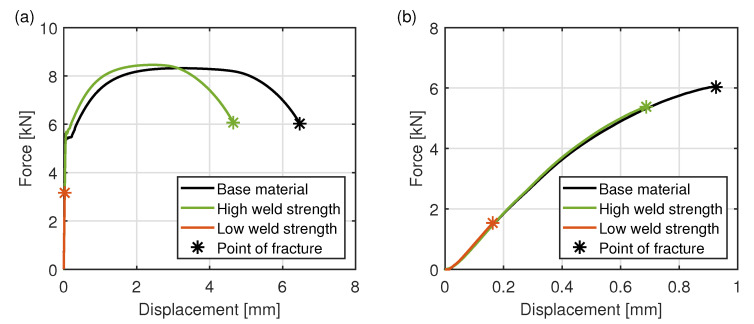
Force-displacement curves of the base material and of the welded specimens with high and low weld strength in the (**a**) tensile test and (**b**) bending test.

Figure 8 shows the force-displacement curves obtained in the tensile and bending tests for the base material and the welded specimens. The force-displacement curve of the welded specimen with the highest measured force is labeled as “high weld strength” in the diagram. The “low weld strength” shows the force-displacement curve of the welded specimen, which is broken at the lowest fracture force. In the tensile test, the maximum force is almost identical for the base material and the specimen with the high weld strength. However, the elongation at break is about 25% lower for the welded specimen due to the influence of the hard weld seam, whereas the welded specimen with the lowest weld strength breaks already after a displacement of 0.03 mm and at breaking forces of about 35% of the base material. In the bending test, the base material breaks at an about 10% higher force level than the specimen with the high weld strength, while the welded specimen with the lowest weld strength breaks at about 25% of the force of the base material.

**Figure 9:**
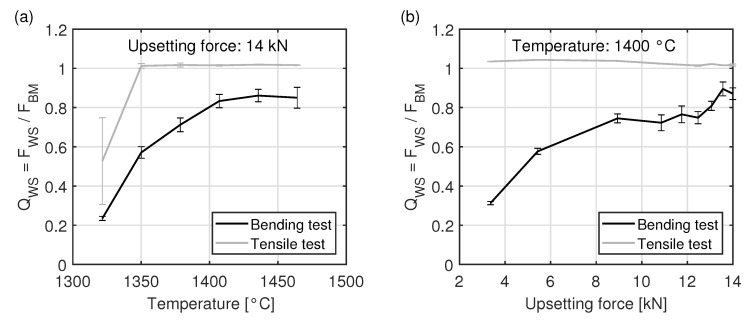
Dependence of the weld seam strength ratio $Q_{\mathrm{WS}}$ (**a**) on the temperature at a constant upsetting force and (**b**) on the upsetting force at a constant temperature.

The dependence of the weld seam strength ratio $Q_{\mathrm{WS}}$ on temperature and upsetting force, established with the welded tensile specimens and hardened bending specimens, is shown in Figure 9. In order to calculate the weld seam strength ratio $Q_{\mathrm{WS}}$ of the fracture forces according to (3), five specimens of the base material and two specimens with weld seam were tested for each welding parameter setting. Thereby, fracture forces of the base material $F_{\mathrm{BM}}$ in the tensile test of 8319 ± 8 N and in the bending test of 6016 ± 162 N were obtained. The calculation of the standard deviation

(4) $$\sigma_{Q}=\sqrt{\frac{\sigma_{\mathrm{WS}}^{2}}{F_{\mathrm{BM}}^{2}}+\frac{\sigma_{\mathrm{BM}}^{2}F_{\mathrm{WS}}^{2}}{F_{\mathrm{BM}}^{4}}}$$

of the weld seam strength ratio $Q_{\mathrm{WS}}$ was performed with the propagation of uncertainty. Where $\sigma_{\mathrm{BM}}$ is the standard deviation of the fracture force from the base material $F_{\mathrm{BM}}$ and $\sigma_{\mathrm{WS}}$ is the standard deviation of the fracture force of the specimens with weld seam $F_{\mathrm{WS}}$.

The weld seam strength ratio $Q_{\mathrm{WS}}$, obtained in the tensile test, is about 0.5 at a welding temperature of 1320 °C. Thereby, the standard deviation of 0.22 is very large, since the investigated breaking forces show a strong variation for this welding parameter. However, the weld seam strength ratio $Q_{\mathrm{WS}}$ for all other welding parameters, investigated in the tensile test, is slightly above 1. As shown in Figure 7, these specimens all fracture adjacent to the weld seam. Due to the constant fracture location, the investigated standard deviations are close to zero. Since almost all specimens fracture outside the hardened weld seam, it is not possible to establish a correlation between the welding parameters and the weld seam strength ratio $Q_{\mathrm{WS}}$ in the tensile test. Therefore, only the bending test will be considered in the following.

The weld seam strength ratio $Q_{\mathrm{WS}}$, obtained in the bending test on hardened specimens, increases with increasing temperature and upsetting force. In a range from 1320 °C to 1410 °C the measured weld seam strength ratio $Q_{\mathrm{WS}}$ increases from 0.22 to 0.85. However, above 1410 °C no further increase of $Q_{\mathrm{WS}}$ can be observed at a constant upsetting force of 14 kN. At a temperature of 1400 °C, a weld seam strength ratio of $Q_{\mathrm{WS}}$ = 0.75 is reached at an upsetting force of 9 kN. At this temperature, the weld seam strength ratio $Q_{\mathrm{WS}}$ remains almost constant in a range from 9 kN to 12 kN. A further increase of the weld seam strength up to about 0.9 occurs only with a further increase in the upsetting force. This is the highest value of the weld seam strength ratio that has been obtained in this study.

In order to transfer the relationship of the weld seam strength ratio $Q_{\mathrm{WS}}$ to other sheet thicknesses, it is necessary to use the contact normal stress instead of the upsetting force. For this reason, the correlation between the upsetting force and the contact normal stress was established simulatively with the FEM software MSC Marc/Mentat^®^ 2019 (MSC Software Corporation, Newport Beach, CA, USA). Therefore, FEM simulations of the press welding for temperatures from 1300 °C to 1500 °C and upsetting forces from 2 kN to 15 kN were carried out. The simulation was based on elastic-plastic material behavior. For this purpose, flow curves as a function of strain, strain rate and temperature were measured on a Gleeble^®^ 3800-GTC (Dynamic Systems Inc., Poestenkill, NY, USA) [25]. Based on the simulation results, the contact normal stress $p_{c}$ for different temperatures *T* and upsetting forces $F_{c}$ can be described in terms of a linear model

(5) $$p_{c}=a+bF_{c}+cT$$

with *a* = 45.04 MPa, *b* = $6.26\;\times \;10^{-3}$ MPa/N and *c* = $-2.53\;\times \;10^{-2}$ MPa/°C. However, the calculation of the contact normal stress $p_{c}$ using the relation $p_{c}=F_{c}/A$ would also be possible, where *A* is the cross section area of the specimen. However, this formula overestimates the contact normal stress up to 30%, since the increase of the contact area due to the deformation of the band edges is not taken into account.

**Figure 10:**
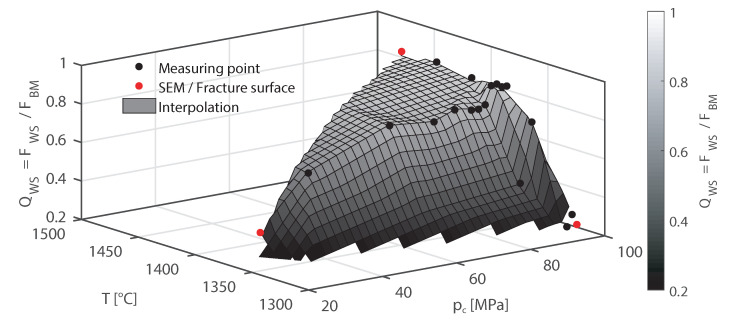
Dependence of the weld seam strength ratio $Q_{\mathrm{WS}}$ on temperature *T* and contact normal stress $p_{c}$, established in the bending test with hardened specimens.

With the correlation given in (5), the relationship between contact normal stress, temperature and weld seam strength ratio $Q_{\mathrm{WS}}$ shown in Figure 10 is obtained. A weld seam strength ratio $Q_{\mathrm{WS}}$ of greater than 0.8 is achieved at temperatures greater than 1400 °C and with contact normal stresses greater than 60 MPa.

### 3.3. Relationship between Weld Seam Strength Ratio $Q_{\mathrm{WS}}$ and Oxide Inclusions

In order to establish a relationship between the measured mechanical weld seam strength ratios, the microstructure in the bonding line and the fracture surface, three different welding parameters and the base material in the hardened state were compared. Table 7 shows SEM images of the base material, a weld seam with high mechanical strength and a weld seam made with low upsetting pressure or low welding temperature. The specimens were etched with a modified Beraha etchant for 20 s. In the condition with $Q_{\mathrm{WS}}=0.88$ the bonding line is no longer visible after hardening. Due to low temperatures ($Q_{\mathrm{WS}}=0.22$) or low upsetting forces ($Q_{\mathrm{WS}}=0.33$), oxides remain in the bonding line. At high temperatures and low upsetting pressure, the band edges are melted, but the upsetting force is not high enough to squeeze the melt including the oxides out of the welding zone. If the temperature is too low ($Q_{\mathrm{WS}}=0.22$) the band edges are not melted. As a result, even higher upsetting pressures can be not sufficient to deform the band edges plastically and squeeze the oxides out of the welding zone. In both cases, oxides are present in the bonding line after solidification. In the welded plate with low upsetting force ($Q_{\mathrm{WS}}=0.33$) these oxides are distributed over a narrow area of about 4 μm width. In the welded plate with low temperature ($Q_{\mathrm{WS}}=0.22$), the oxides are smaller and distributed over a large width of 25 μm.

The oxides in the weld seam form imperfections and lead to initial failure of the hardened bending specimens in the bonding line (Table 8). The fracture surface of the hardened base material has a pronounced topography and is a mixture of brittle and ductile fracture. The welded bending specimen with high weld seam strength ratio ($Q_{\mathrm{WS}}=0.88$) fractures in the bonding line. The fracture surface has areas of brittle and ductile fracture like the base material. In contrast to the base material, however, some oxides are visible on the fracture surface. These single oxides are the reason why the weld seam strength ratio is slightly lower compared to the base material. The two specimens with the low weld seam strength ratio exhibit a very smooth, mostly ductile fracture surface. The dimples of the ductile fracture are much smaller than in the base material and oxides can be found in the majority of the dimples. EDS analysis has revealed that these oxides consist of manganese, silicon, iron and aluminium. During bending, stress concentrations separate oxide inclusions from the metal matrix and generate micro voids. This voids increase in size during the deformation process and lead to fracture by connecting together [37,38]. A high amount of oxides in the bonding line leads to the formation of many micro voids. During deformation, these voids quickly link together and thus lead to a lower strength of the bonding line compared to the base material.

## 4. Discussion

In tensile tests, the specimens in the welded state break almost always outside the hardened weld seam. This behavior has already been reported [17,34,35]. Thereby, the measured forces are often larger than those of the base material [36]. It is argued that the reason for the fracture next to the weld seam is due to the strongly different yield strengths of the steel in the normalized and hardened state. As soon as the yield strength is reached by the base material next to the weld seam, necking starts there. Afterwards, the tensile force and thus the load on the weld seam decreases. Thus, from this point on, only the base material is tested. For this reason, in the case of longitudinally welded tubes with diameter >150 mm, tensile specimens are also sometimes manufactured with the weld seam parallel to the tensile direction [39]. In this case, the weld seam, the heat-affected zone and the base material are tested together. Thereby, the investigation of the weld seam strength requires an elaborate calculation based on the rule of mixtures [40,41]. In addition, a comparison with the base material is difficult due to the specimen design. Furthermore, this type of specimen can not be used for tubes with a diameter <150 mm. Therefore, tensile tests on tubular specimens in the welded condition are not suitable for a systematic investigation of the weld seam strength ratio $Q_{\mathrm{WS}}$.

The experimental electro-thermo-mechanical simulator for HFI welding presented in this paper allows the selective variation of the welding temperature and the contact normal stress. The specimens welded with this simulator were tested in the hardened condition using a four-point bending test. Thus, a correlation between the welding temperature, the contact normal stress and the weld seam strength ratio could be established. In the investigated process window, the weld seam strength ratio increases continuously in the temperature range from 1320 °C to 1410 °C and in the contact normal stress range from 30 MPa to 60 MPa. However, at temperatures above 1410 °C and contact normal stresses above 60 MPa, a plateau is formed for the weld seam strength ratio. Consequently, a further increase of the temperature up to 1500 °C and the contact normal stress up to 100 MPa does not lead to a further significant increase of the weld seam strength ratio.

According to [42] the bonding line in HFI weldments is weakened due to oxide formations and the presence of inclusions or voids. Based on SEM images in Table 7 and examination of the fracture surfaces (Table 8), it was shown that the number of oxides in the bonding line increases with decreasing temperature and decreasing upsetting pressure. Some oxides are also present on the fracture surfaces with the highest measured weld seam strength ratio. Therefore, the fracture occurs predominantly in the bonding line. In accordance with this finding, all specimens in the hardened state broke in the bonding line in the performed four-point bending tests. In the hardened state however, the mechanical material properties are nearly the same in the bonding line, the heat affected zone and the base material. In addition, in the four-point bending test the area around the weld seam is subjected to maximum load. Due to the applied load in the four-point bending test, the uniform material properties in the hardened state and the weakening of the bonding line by oxides, it is assumed that the fracture strength of the specimens with weld seam will always be less than that of the base material.

The maximum weld seam strength ratio $Q_{\mathrm{WS}}$ achieved for the 34MnB5 steel used in this study is 0.90. This value is comparable to the weld joint efficiency coefficient which is used in strength calculations to account the weakening due to the weld seam by Rosenfeld [43]. In case of resistance welded tubes made of low carbon steel, the joint efficiency coefficient was established to be 0.85 [44]. In this study, values of $Q_{\mathrm{WS}}$ > 0.85 were obtained for welding temperatures larger than 1410 °C and contact normal stresses larger than 90 MPa. Kannan et al. [16] obtained a similar temperature range for resistance welding in their study on the bond formation of X70 pipeline steel using a Gleeble^®^ simulator. The range of the welding temperatures and the weld seam strength ratio $Q_{\mathrm{WS}}$ determined in this study are therefore within the expected range.

A comparison of the obtained weld seam strength ratio $Q_{\mathrm{WS}}$ with scanning electron microscopy images and the fracture surfaces shows that the weld seam strength ratio $Q_{\mathrm{WS}}$ decreases with increasing oxide inclusions in the bonding line. These oxides in the bonding line have been reported as a primary factor for cracks in other studies [20,30]. They form at high temperatures at the band edges before welding [45]. Due to the partial melting of the band edges and the high contact normal stress, inclusions are normally squeezed out of the weld seam with the molten and semi-molten metal. If the contact pressure is too low, the oxide removal is incomplete and thus oxide residues remain in the bonding line. If the welding temperature is too low, even high contact normal stresses are not sufficient to deform the weld seam to squeeze out all the oxides.

In this study, it was not possible to increase the contact normal stress above 100 MPa. However, it is assumed that a higher contact normal stress does not lead to any further increase in the weld seam strength ratio $Q_{\mathrm{WS}}$. The highest welding temperature studied in the experiments was 1500 °C. The authors assume that the weld seam strength ratio $Q_{\mathrm{WS}}$ at higher temperatures will remain at a value of 0.90 until weld spatter forms due to excessive temperatures. For other steel grades, a similar relationship of welding temperature, contact normal stress and weld seam strength ratio $Q_{\mathrm{WS}}$ can be assumed. However, it is expected that the maximum achievable weld seam strength ratio $Q_{\mathrm{WS}}$ will vary for other steel grades.

## 5. Conclusions

In this study, the influence of welding temperature and contact normal stress on the weld seam strength ratio of high frequency induction longitudinally welded steel tubes was investigated. The obtained weld seam strength ratio was correlated with the microstructure of the weldments. The following conclusions can be drawn:

- A minimum welding temperature of 1320 °C and a minimum contact normal stress of 30 MPa are necessary in order to join two 34MnB5 steel sheets. At lower welding temperatures or contact normal stresses, the two steel sheets cannot be joined by HFI welding.
- As a result of the welding process, the specimens consist of a hard weld seam and the base material with low hardness. It has been observed that the specimens in that form usually break in the base material. For this reason, the welded specimens must be hardened before the mechanical test in order to be able to reliably obtain the weld seam strength ratio. Hardening the specimens also leads to uniform hardness values and grain sizes in the bonding line, the heat affected zone and the base material. In addition, the bainitic ferrite formed in the bonding line during HFI welding is transformed into a fully martensitic structure during hardening.
- The weld seam strength ratio $Q_{\mathrm{WS}}$ of the investigated 34MnB5 steel sheets increases with welding temperature and contact normal stress. At welding temperatures above 1410 °C and contact normal stresses above 60 MPa, a kind of plateau forms for the weld seam strength ratio $Q_{\mathrm{WS}}$. The maximum determined weld seam strength ratio $Q_{\mathrm{WS}}$ is 0.9 for the HFI welded 34MnB5 plates.
- Metallographic examinations of the weld seam and the fracture surfaces have shown that the weld seam strength ratio $Q_{\mathrm{WS}}$ is related to the amount of oxides in the bonding line. Welds with a high weld seam strength ratio $Q_{\mathrm{WS}}$ have very few oxides in the bonding line and on the fracture surface. In contrast, welds with low weld seam strength ratio $Q_{\mathrm{WS}}$ have many oxides in the bonding line and on the fracture surface.

## Figures and Tables

**Table 1:** Chemical composition of investigated manganese-boron steel 34MnB5 in wt%.

C	Si	Mn	P	S	Al	Cr	Ti	Cu	B
0.33	0.23	1.33	0.01	0.001	0.04	0.13	0.03	0.02	0.003

**Table 2:** Light optical microscopy images and prior austenite grain size (PAGs) of the bonding line (BL), coarse and fine grained heat affected zone (CGHAZ, FGHAZ) and the base material (BM) of the weld seam in the as-welded state at different distances (D) from the bonding line.

	BL	CGHAZ	FGHAZ	BM
D	0.0 mm	0.5 mm	1.0 mm	3.0 mm
Light microscopy	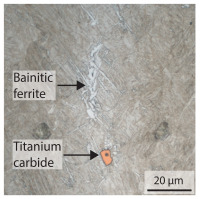	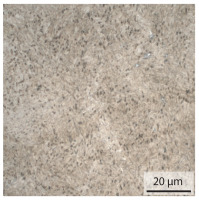	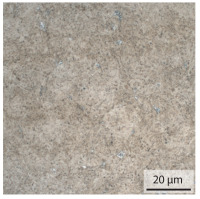	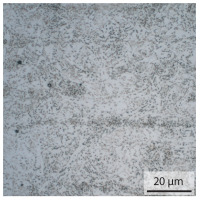
PAGs/Grain size	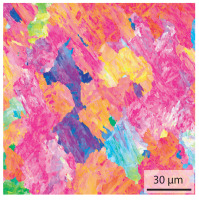	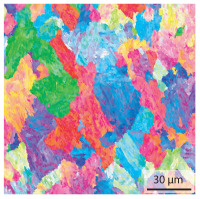	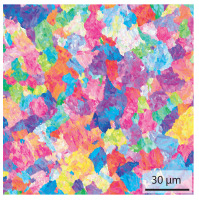	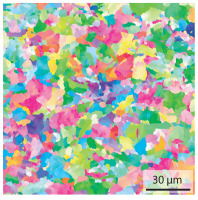

**Table 3:** Analysis of the chemical composition with energy dispersive spectroscopy (EDS) in the base material and in the bonding line in wt%.

Location	C	Si	Mn	Al	Fe
Base material	2.50	0.22	1.37	0.01	95.82
Bonding line	1.58	0.12	1.10	0.00	97.10

**Table 4:** Investigation of the bonding line with SEM, EBSD for differentiation between body-centered cubic (bcc) and face-centered cubic (fcc) phase and nanohardness measurements.

	SEM	SEM (Detail)	Crystal Lattice	Hardness
Bonding line	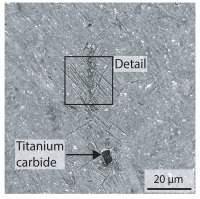	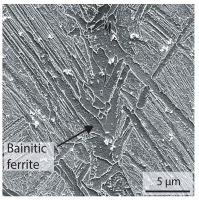	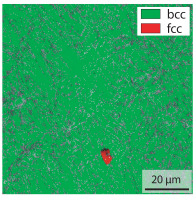	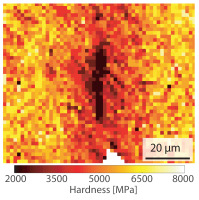

**Table 5:** Light optical microscopy images and prior austenite grain size (PAGs) of the bonding line (BL), coarse and fine grained heat affected zone (CGHAZ, FGHAZ) and the base material (BM) of the weld seam in the hardened state at different distances (D) from the bonding line.

	BL	CGHAZ	FGHAZ	BM
D	0.0 mm	0.5 mm	1.0 mm	3.0 mm
Light microscopy	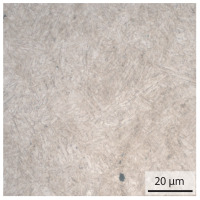	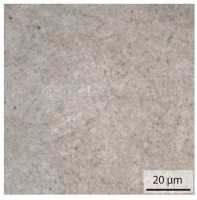	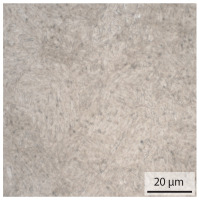	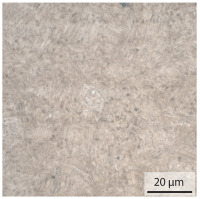
PAGs	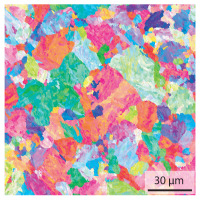	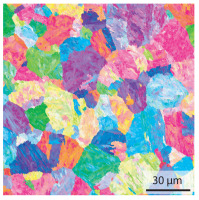	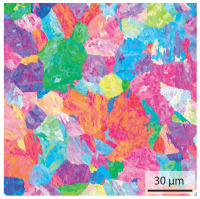	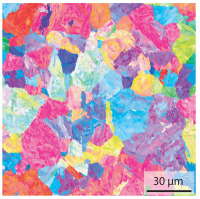

**Table 6:** Power *P*, temperature *T*, upsetting force $F_{c}$, contact normal stress $p_{c}$, fracture force $F_{\mathrm{WS}}$ and weld seam strength ratio $Q_{\mathrm{WS}}$ of the tensile test with as-welded specimens and the bending test with hardened specimens of the different welding settings.

Welding Settings				Tensile Test		Bending Test	
P	T	$F_{c}$	$p_{c}$	$F_{\mathrm{WS}}$	$Q_{\mathrm{WS}}$	$F_{\mathrm{WS}}$	$Q_{\mathrm{WS}}$
[kW]	**[°C]**	**[kN]**	**[MPa]**	**[N]**	**[-]**	**[N]**	**[-]**
96	1464	14.0	95.6	8455 ± 6	1.02 ± 0	5116 ± 261	0.85 ± 0.05
93	1436	14.0	96.3	8476 ± 16	1.02 ± 0	5181 ± 35	0.86 ± 0.02
90	1407	14.0	97.0	8446 ± 26	1.02 ± 0	5013 ± 94	0.83 ± 0.03
87	1379	14.0	97.8	8460 ± 83	1.02 ± 0.01	4285 ± 145	0.71 ± 0.03
87	1379	13.8	96.4	8504 ± 1	1.02 ± 0	4956 ± 146	0.82 ± 0.03
87	1379	13.6	95.0	8448 ± 14	1.02 ± 0	4886 ± 196	0.81 ± 0.04
87	1379	13.3	93.5	8501 ± 9	1.02 ± 0	5153 ± 101	0.86 ± 0.03
87	1379	13.1	91.9	8502 ± 10	1.02 ± 0	4866 ± 46	0.81 ± 0.02
87	1379	12.8	90.1	8497 ± 3	1.02 ± 0	4710 ± 383	0.78 ± 0.07
87	1379	12.5	88.2	8437 ± 25	1.01 ± 0.01	4503 ± 142	0.75 ± 0.03
87	1379	11.8	83.7	8472 ± 1	1.02 ± 0	4605 ± 224	0.77 ± 0.04
87	1379	10.9	78.1	8517 ± 16	1.02 ± 0	4348 ± 214	0.72 ± 0.04
87	1379	8.9	66.1	8635 ± 8	1.04 ± 0	4482 ± 66	0.75 ± 0.02
87	1379	5.4	44.2	8679 ± 11	1.04 ± 0	3470 ± 57	0.58 ± 0.02
87	1379	3.4	31.2	8608 ± 5	1.03 ± 0	1882 ± 98	0.31 ± 0.01
84	1350	14.0	98.5	8423 ± 48	1.01 ± 0.01	3436 ± 63	0.57 ± 0.02
84	1350	13.6	95.8	8423 ± 97	1.01 ± 0.01	3436 ± 125	0.57 ± 0.03
84	1350	13.1	92.6	8477 ± 27	1.02 ± 0.01	2437 ± 370	0.40 ± 0.06
81	1322	14.0	99.2	4384 ± 1840	0.53 ± 0.22	1413 ± 35	0.23 ± 0.01
81	1322	13.8	97.9	8476 ± 8	1.02 ± 0	1663 ± 0	0.28 ± 0.01
81	1322	13.6	96.5	1625 ± 1150	0.20 ± 0.14	1414 ± 35	0.23 ± 0.01
78	1293	14.0	99.9	0 ± 0	0.00 ± 0	0 ± 0	0.00 ± 0
78	1293	13.6	97.2	0 ± 0	0.00 ± 0	0 ± 0	0.00 ± 0

**Table 7:** Comparison of SEM images of the hardened base material and the bonding line of hardened 34MnB5 steel sheets welded with different welding parameters (*T*, $p_{c}$) and different weld seam strength ratios $Q_{\mathrm{WS}}$.

	$Q_{\mathrm{WS}}=1.00$	$Q_{\mathrm{WS}}=0.88$	$Q_{\mathrm{WS}}=0.33$	$Q_{\mathrm{WS}}=0.22$
	Base	T= 1465 °C	T= 1380 °C	T= 1320 °C
	material	$p_{c}=$ 95 MPa	$p_{c}=$ 30 MPa	$p_{c}=$ 97 MPa
SEM	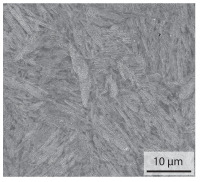	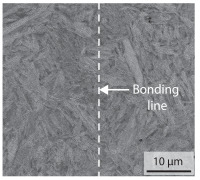	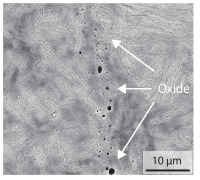	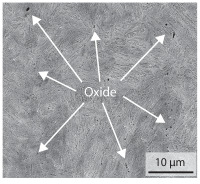

**Table 8:** Comparison of the fracture surfaces of the hardened bending specimens of 34MnB5 steel sheets welded with different welding parameters (*T*, $p_{c}$) and different weld seam strength ratios $Q_{\mathrm{WS}}$.

	$Q_{\mathrm{WS}}=1.00$	$Q_{\mathrm{WS}}=0.88$	$Q_{\mathrm{WS}}=0.33$	$Q_{\mathrm{WS}}=0.22$
	Base	T= 1465 °C	T= 1380 °C	T= 1320 °C
	material	$p_{c}=$ 95 MPa	$p_{c}=$ 30 MPa	$p_{c}=$ 97 MPa
Overview	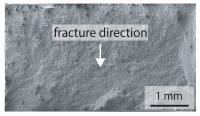	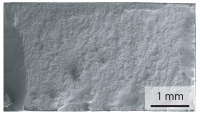	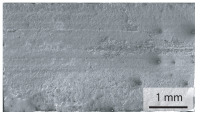	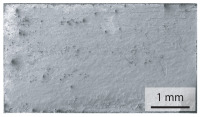
×2500	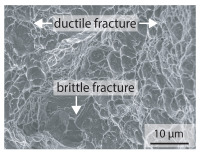	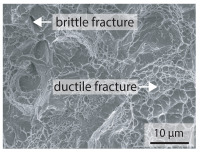	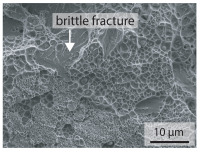	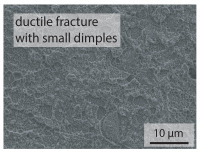
×10000	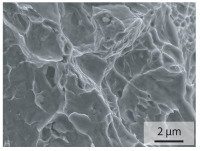	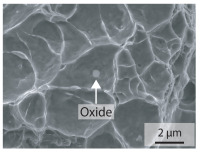	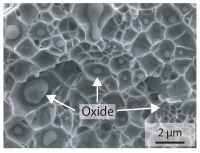	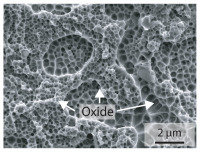

## Data Availability

The data generated in this study cannot be shared at this time. They may be available from the corresponding author upon reasonable request.
